# The Impact of Cornelian Cherry (*Cornus mas* L.) on Cardiometabolic Risk Factors: A Meta-Analysis of Randomised Controlled Trials

**DOI:** 10.3390/nu16132173

**Published:** 2024-07-08

**Authors:** Oleg Frumuzachi, Helena Kieserling, Sascha Rohn, Andrei Mocan, Gianina Crișan

**Affiliations:** 1 Department of Pharmaceutical Botany, Faculty of Pharmacy, “Iuliu Hațieganu” University of Medicine and Pharmacy, 23 Gheorghe Marinescu Street, 400337 Cluj-Napoca, Romania; oleg.frumuzachi@elearn.umfcluj.ro (O.F.); gcrisan@umfcluj.ro (G.C.); 2 Department of Food Chemistry and Analysis, Institute of Food Technology and Food Chemistry, Technische Universität Berlin, Gustav-Meyer-Allee 25, 13355 Berlin, Germany; helena.schestkowa@tu-berlin.de (H.K.); rohn@tu-berlin.de (S.R.); 3 Research Centre of Medicinal and Aromatic Plants, “George Emil Palade” University of Medicine, Pharmacy, Sciences and Technology of Targu Mures, 38 Gheorghe Marinescu Street, 540139 Targu Mures, Romania

**Keywords:** lipid profile, weight loss, glucose metabolism, anthocyanins, metabolic disease

## Abstract

This meta-analysis aimed to summarise clinical evidence regarding the effect of supplementation with cornelian cherry (*Cornus mas* L.) on different cardiometabolic outcomes. An extensive literature survey was carried out until 10 April 2024. A total of 415 participants from six eligible studies were included. The overall results from the random-effects model indicated that cornelian cherry supplementation significantly reduced body weight (standardised mean difference [SMD] = −0.27, confidence interval [CI]: −0.52, −0.02, *p* = 0.03), body mass index (SMD = −0.42, CI: −0.73, −0.12, *p* = 0.007), fasting blood glucose (SMD = −0.46, CI: −0.74, −0.18, *p* = 0.001), glycated haemoglobin (SMD = −0.70, CI: −1.19, −0.22, *p* = 0.005), and HOMA-IR (SMD = −0.89, CI: −1.62, −0.16, *p* = 0.02), while high-density lipoprotein cholesterol significantly increased (SMD = 0.38, CI: 0.10, 0.65, *p* = 0.007). A sensitivity analysis showed that cornelian cherry supplementation significantly reduced total plasma triglycerides, total cholesterol, low-density lipoprotein cholesterol, and insulin levels. Cornelian cherry supplementation did not significantly affect waist circumference and liver parameters among the participants. Considering these findings, this meta-analysis indicates that supplementation with cornelian cherry may impact diverse cardiometabolic risk factors among individuals considered to be at a high risk.

## 1. Introduction

Cardiometabolic diseases refer to a group of conditions that involve a combination of cardiovascular and metabolic factors. These diseases frequently exhibit common risk factors, underlying mechanisms, and health implications. The most common risk factors include dyslipidaemia, dysglycaemia, systemic hypertension, and central obesity [1]. Increased liver enzyme levels of, e.g., aspartate aminotransferase and alanine aminotransferase have conventionally been reported as indicators of liver dysfunction, notably in conditions such as non-alcoholic fatty liver disease (NAFLD) [2]. However, recent findings have linked serum aspartate aminotransferase and alanine aminotransferase levels to the development of type 2 diabetes mellitus (T2DM), irrespective of conventional risk factors [3]. Additionally, increased aminotransferase levels have been correlated with established risk factors for cardiometabolic disease, such as elevated blood pressure, body mass index, and fasting blood glucose levels [4]. This finding underscores the potential preventive significance of aminotransferases.

The incidence of cardiometabolic diseases has increased globally due to a range of lifestyle factors, including sedentary behaviour, smoking, and poor dietary choices [5]. As reported by the World Health Organization (WHO), non-communicable diseases (NCDs) are responsible for approximately 17 million deaths annually among individuals under the age of 70 worldwide [6], with a significant portion of these deaths linked to cardiometabolic diseases. Projections from the WHO indicate that, by the year 2030, NCDs will constitute 77% of the overall global disease burden [7].

In this context, lifestyle changes, including increasing the frequency and intensity of physical activity and adopting a healthy diet, constitute the primary therapeutic strategies for addressing cardiometabolic disorders [8,9,10]. The inclusion of fruits and vegetables plays a crucial role. By integrating these into one’s diet, individuals can significantly contribute to managing cardiometabolic disorders [11]. Cornelian cherry (*Cornus mas* L.) fruits have emerged as a complementary intervention to mitigate the risk factors associated with cardiometabolic diseases. Cornelian cherries possess a rich history in traditional medicine across various regions, including the Caucasus, Central Asia, Slovakia, Turkey, Azerbaijan, and Iran, where they have been used for over a millennium to address a range of health issues [12]. These health issues include sore throats, digestion problems, and infectious diseases like measles and chickenpox, as well as liver and kidney ailments [12]. The composition of cornelian cherry is diverse, with its fruits being particularly rich in bioactive compounds such as (poly)phenols (anthocyanins, flavonols, phenolic acids, and tannins), iridoids, and triterpenoids [13]. Several animal studies have demonstrated the potential health benefits of cornelian cherry supplementation, including its antidiabetic, anti-obesity, hypolipidemic, hepatoprotective, and cardioprotective effects [14,15,16,17]. Moreover, a recent meta-analysis exploring the impact of cornelian cherry supplementation on the lipid profiles in rat models concluded that such supplementation resulted in notable reductions in total cholesterol, low-density lipoprotein cholesterol, and total triglycerides levels [18]. These effects have been attributed to the abovementioned bioactive compounds present in cornelian cherry, underscoring the potential of cornelian cherry as a dietary supplement or therapeutic agent in managing various cardiometabolic conditions. Therefore, supplemented forms can offer convenience and potentially concentrated doses of beneficial nutrients found in raw cornelian cherries [19].

Considering its bioactive composition, promising results from animal studies, and the rich diversity of ethnopharmacological uses of cornelian cherry, several human randomised controlled trials (RCTs) have evaluated how supplementation with cornelian cherry affects various cardiometabolic risk factors. These trials have highlighted the potential benefits of cornelian cherry fruits in reducing the risk factors linked to cardiometabolic diseases. However, until now, no meta-analysis has assessed the overall impact of cornelian cherry supplementation across these trials. Therefore, the aim of this study was to perform a meta-analysis to assess the data from RCTs regarding the supplementation of cornelian cherry fruit, powder, and extract on cardiometabolic risk factors in adult participants aged 18 years and older. The evaluation focused on primary outcomes including low-density lipoprotein cholesterol (LDL-C), high-density lipoprotein cholesterol (HDL-C), total cholesterol (TC), triglycerides (TG), systolic blood pressure (SBP), diastolic blood pressure (DBP), fasting blood glucose (FBG), and body mass index (BMI). Secondary outcomes included body weight (BW), waist circumference (WC), insulin levels, glycated haemoglobin (HbA1c), homeostatic model assessment of insulin resistance (HOMA-IR), aspartate aminotransferase (AST), and alanine aminotransferase (ALT). Both these primary and secondary selected outcomes are typically influenced by diet quality (e.g., the intake of saturated fats, sugars, and fibre), physical activity levels, smoking habits, alcohol consumption, stress management, and sleep patterns.

## 2. Methods

The meta-analysis was conducted in accordance with the *Preferred Reporting Items for Systematic Reviews and Meta-Analyse* (PRISMA-2020) guidelines reported by Page et al. [20]. Moreover, the meta-analysis was registered in the International Prospective Register of Systematic Reviews (PROSPERO: CRD42024528587) prior to beginning.

### 2.1. Literature Search

A systematic search across the prominent databases *Embase*, *Scopus*, *PubMed*, and *Web of Science* was carried out until 10 April 2024, without applying any filters. The following search query “(cornus mas OR cornelian cherry) AND trial” was utilised. Two researchers independently conducted all phases of the systematic review process. Initially, the titles and abstracts of the retrieved articles underwent eligibility screening. The full texts of the relevant studies were then assessed for inclusion, with any disagreements between the researchers being resolved through discussion and consensus. Language restrictions were not imposed. Furthermore, the reference lists of eligible RCTs were screened to identify additional relevant studies.

### 2.2. Eligibility Criteria

The comprehensive *Population, Intervention, Comparison, Outcomes and Study* (PICOS) selection criteria are presented in Table 1. The inclusion criteria for the studies were: (i) supplementation with cornelian cherry fruit, powder or extract as part of the intervention; (ii) an RCT with either a crossover or parallel trial design, with a minimum duration of ≥2 weeks; (iii) the inclusion of adult participants (aged ≥ 18 years), excluding pregnant individuals, whether healthy or otherwise; and (iv) evaluation of the effects of cornelian cherry on cardiometabolic risk factors, including BW, BMI, WC, TG, TC, LDL-C, HDL-C, SBP, DBP, FBG, insulin, HbA1c, HOMA-IR, AST, and ALT. Studies were excluded when they: (i) did not have a control group; (ii) were not randomised and/or the duration of the study was <2 weeks; (iii) were conducted on individuals <18 years old or included pregnant women; and (iv) did not evaluate any of the mentioned outcomes. Moreover, trial protocols, observational studies, case reports, case series, in vitro studies, animal experiments, and abstracts without findings were not included in the meta-analysis.

The inclusion criteria for the studies were carefully chosen to ensure the relevance and quality of the meta-analysis: (i) cornelian cherry is known for its bioactive compounds, which have shown potential benefits in previous studies, including studies that used cornelian cherry in various forms (powder or extract), ensuring a comprehensive evaluation of its effects on cardiometabolic risk factors; (ii) RCTs are considered to be the gold standard for clinical research because they reduce bias and allow for causal inferences. A threshold of ≥2 weeks was chosen based on previous meta-analyses and clinical studies that have indicated that a minimum duration of two weeks is necessary to observe significant changes in cardiometabolic risk factors with dietary interventions [21]; (iii) adults are the primary demographic for studying cardiometabolic risk factors, as they are more likely to be affected by these conditions. Pregnant individuals were excluded to avoid confounding factors related to pregnancy, such as hormonal changes and altered metabolic states, which could affect the outcomes; and (iv) these specific cardiometabolic risk factors were chosen because they are widely recognised indicators of cardiometabolic health and are commonly assessed in clinical trials.

The rationality behind the exclusion criteria were: (i) a control group is essential to determine the effect of an intervention. Studies without a control group cannot provide a valid comparison, making it impossible to attribute the observed effects to cornelian cherry supplementation alone; (ii) randomisation minimises selection bias and ensures that the results are due to the intervention rather than other factors. As previously mentioned, a duration of at least 2 weeks is necessary to observe meaningful changes in cardiometabolic risk factors; (iii) including only adults ensures that the results are applicable to the population most at risk for cardiometabolic diseases. Excluding pregnant women eliminates the confounding effects of pregnancy on cardiometabolic risk factors; and (iv) the focus of this meta-analysis is on specific cardiometabolic risk factors. Studies not evaluating these outcomes do not contribute relevant data to the analysis. Moreover, trial protocols, observational studies, case reports, case series, in vitro studies, animal experiments, or abstracts without findings were not included in the meta-analysis, because they do not provide the same level of evidence as RCTs and often lack the necessary data to assess the impact of cornelian cherry supplementation on cardiometabolic risk factors.

### 2.3. Data Extraction

Two of the authors independently conducted an initial screening of the article titles and abstracts retrieved from the online database searches. Subsequently, all pertinent data were extracted and cross-verified by other authors. The extracted data included the first author’s name, publication year, study location, design, participants’ health status, dose and intervention type, duration, total number of subjects included in the study, and reported outcomes. Additionally, correspondence was initiated with the corresponding authors of specific RCTs to obtain any missing data that were not originally documented in the articles.

### 2.4. Quality Assessment

Two of the authors independently assessed the methodological quality of the eligible studies using the revised Cochrane risk-of-bias tool for randomised trials (RoB 2) [22,23]. RoB 2 is organised into a predefined set of bias domains (bias arising from the randomisation process, bias due to deviations from intended interventions, bias due to missing outcome data, bias in the measurement of the outcome, and bias in the selection of the reported result), which address various aspects of trial design, implementation, and reporting. Each domain comprises a series of questions (‘signalling questions’) aimed at gathering information pertinent to the risk of bias. An algorithm generates a proposed judgment regarding the risk of bias for each domain, based on responses to the signalling questions. These judgments may indicate a ‘low’ or ‘high’ risk of bias, or may raise ‘some concerns’. Any discrepancies regarding the methodological quality of the eligible studies were discussed and resolved by consensus.

### 2.5. Data Synthesis and Statistical Analysis

The statistical analysis was performed using RevMan version 5.4 software and R studio 2023.12.1+402 version. A random-effects meta-analysis model was chosen due to the variations in cornelian cherry supplementation among the studies included. This model aimed to evaluate the mean differences in the treatment effect of cornelian cherry supplementation on cardiometabolic outcomes. When data were presented in differing formats, standard equations were applied to derive the mean and standard deviation (SD). For instance, when the SD of the change was unspecified in the studies, it was calculated using the following Formula (1):

SD of change = √((SD_baseline_)^2^ + (SD_end_)^2^ − (2 × R × SD_baseline_ × SD_end_)) (1)

where R = 0.5. Additionally, in trials that provided only the standard error of the mean (SEM), the SD was determined using the Formula (2):

SD = SEM × √n(2)

where “n” is the number of subjects in each group. Furthermore, in studies reporting only the median and 25th–75th percentiles, based on the assumption of normal distribution, the mean and SD were calculated using equations outlined by Wan et al. [24]. Specifically, the mean was estimated using Equation (3):

(q1 + m + q3)/3(3)

where q1 represents the first quartile, q3 represents the third quartile, and m represents the median. The SD approximation was calculated using Equation (4):

(q3 − q1)/η(n)(4)

where η(n) equals 2E(Z(3Q + 1)), with its value depending on the sample size of each arm of the trials.

The outcomes were combined as standardised mean differences (SMD) with 95% confidence intervals (CI), accordingly [25]. Moreover, the extent of statistical heterogeneity among the studies was evaluated using the I^2^ statistic [26]. Statistical heterogeneity was considered to be significant when it ranged from moderate to substantial (I^2^ > 50%) or when there was inconsistency across the RCTs regarding the direction of effect. It was opted not to perform a subgroup analysis due to the limited number of studies included in the meta-analysis and the absence of a definitive dichotomising factor between these studies, such as dosage, administration form, study duration, etc. However, a sensitivity analysis was conducted to evaluate the influence of each study on the overall SMD. Additionally, the potential presence of publication bias using the formal Egger’s test was assessed [27].

## 3. Results

### 3.1. Study Selection

**Figure 1:**
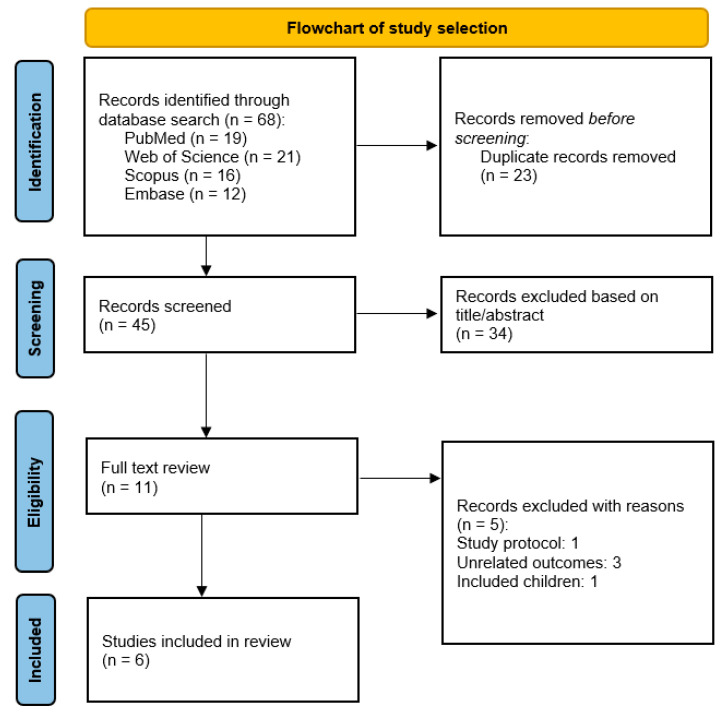
The PRISMA flowchart depicting the study selection process.

Figure 1 illustrates the process of the literature survey and selection. Briefly, during the initial literature survey, a total of 68 articles were identified (Embase = 12, Scopus = 16, PubMed = 19, and Web of Science = 21). After removing duplicates (n = 23), 45 articles remained for screening based on their titles and abstracts. Subsequently, 34 irrelevant articles were excluded (including in vitro and animal studies, review articles, and those with unrelated outcomes), leaving 11 relevant articles for full-text screening. Finally, five articles were excluded as they did not meet the inclusion criteria (three due to unrelated outcomes, one study protocol, and one involving children), while six articles were found to be eligible for inclusion in the meta-analysis.

### 3.2. Study Characteristics

Table 2 provides a summary of the characteristics of the included studies. These studies were published between 2014 [28] and 2024 [29] and were conducted in Iran (four studies, [28,30,31,32]) and Turkey (two studies, [29,33]). All studies included participants of both genders, with a total of 415 participants (209 in the intervention groups and 206 in the placebo groups; 125 males and 290 females). Among these, three studies enrolled participants with metabolic-dysfunction-associated fatty liver disease (MAFLD), a term that was proposed by Eslam et al. [34] to describe fatty liver disease associated with systemic metabolic dysregulation, replacing the previous designation of NAFLD [29,31,32]. One study enrolled participants with T2DM [28], one study enrolled participants with insulin resistance [33], and one study enrolled postmenopausal women [30]. The dosage of cornelian cherry supplementation varied across studies, ranging from 500 mg/day *Cornus mas* (CM) extract (150 mg anthocyanins/day) [28] to 20 mL/day CM extract (equivalent to 2800 mg dried extract, containing 32 mg anthocyanins) [31,32] and 20–30 g/day lyophilised dried CM powder [29,33]. The intervention duration ranged from 6 [28] to 12 weeks [31,32,33]. Among the studies included, four evaluated the impact of CM supplementation on TG [28,29,30,33], three on TC [29,30,33], three on LDL-C [29,30,33], three on HDL-C [29,30,33], four on FBG [28,29,30,33], four on insulin [28,29,30,33], three on HbA1c [28,29,33], three on HOMA-IR [29,30,33], three on ALT [28,29,31], three on AST [28,29,31], four on BW [29,30,32,33], four on BMI [28,29,30,33], and four on WC [29,30,32,33].

### 3.3. Risk of Bias Assessment

**Figure 2:**
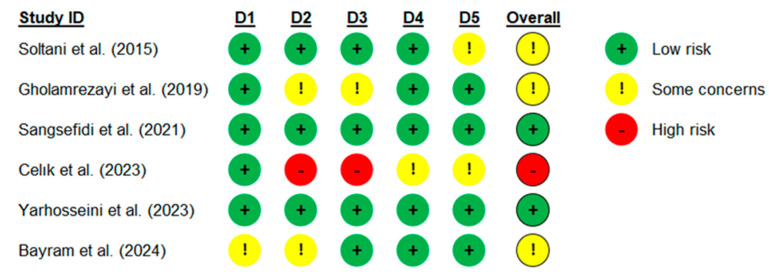
The evaluation of risk of bias concerning each aspect across the studies included in the meta-analysis. D1—randomisation process, D2—deviations from intended interventions, D3—missing outcome data, D4—measurement of the outcome, and D5—selection of the reported results [28,29,30,31,32,33].

**Figure 3:**
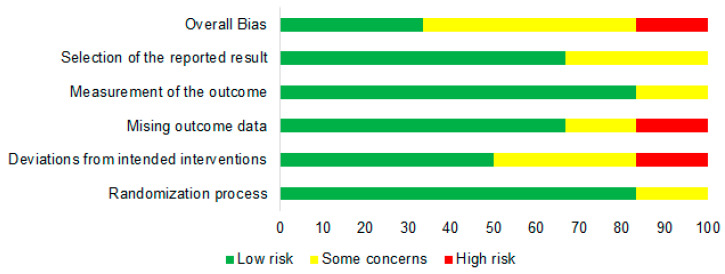
Risk of bias across individual elements, presented as percentage (intention-to-treat), for studies included in the meta-analysis.

The RoB 2 tool, as described by Sterne et al. [22], was employed to evaluate bias across the six randomised clinical trials included in the current meta-analysis. The results of this evaluation are depicted in Figure 2 and Figure 3. The assessment included five domains aimed at evaluating the quality of the included studies: the randomisation process, deviations from intended interventions, missing outcome data, measurement of the outcome, and selection of the reported results. In the domain of the randomisation process, one study raised some concerns; regarding deviations from intended interventions, two studies raised some concerns and one study exhibited a high risk of bias; concerning missing outcome data, one study raised some concerns, while one study had a high risk of bias; regarding the measurement of the outcome, one study raised some concerns; and lastly, in the domain of selection of the reported results, two studies raised some concerns. Overall, utilising the RoB 2 tool, which offers a framework for evaluating the risk of bias, two studies demonstrated a low risk of bias, three studies raised some concerns, while one study exhibited a high risk of bias.

### 3.4. Meta-Analysis Results

#### 3.4.1. Effect of Cornelian Cherry Supplementation on BW, BMI, and WC

**Figure 4:**
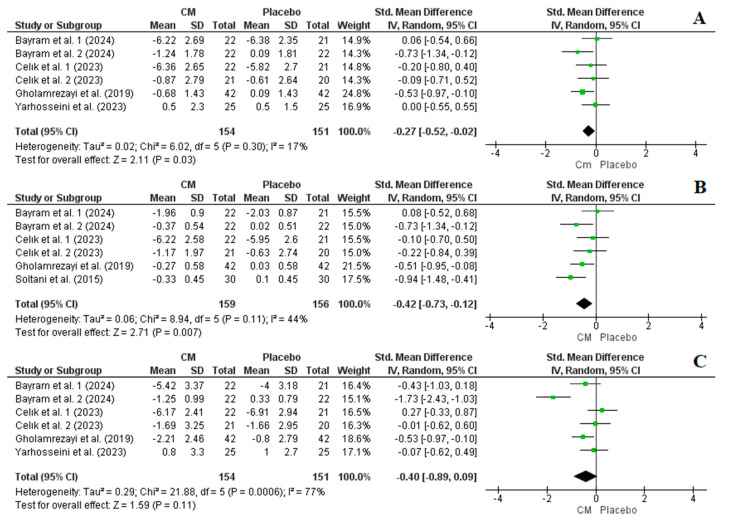
Forest plot representation of RCTs exploring the impact of cornelian cherry supplementation on anthropometric measurements ((**A**): body weight, (**B**): BMI, and (**C**): waist circumference) [28,29,30,32,33].

Four studies, comprising six arms in total, provided data on BW, BMI, and WC as outcome measures. Data for BW and WC were available for 305 participants (154 in the intervention group and 151 in the placebo group), while data for BMI were reported for 315 participants (159 in the intervention group and 156 in the placebo group). Overall, the results from the random-effects model indicated that cornelian cherry supplementation significantly reduced BW (SMD = −0.27, CI: −0.52, −0.02, *p* = 0.03, I^2^ = 17%) and BMI (SMD = −0.42, CI: −0.73, −0.12, *p* = 0.007, I^2^ = 44%), while it did not affect WC (SMD = −0.40, CI: −0.89, 0.09, *p* = 0.11, I^2^ = 77%) (Figure 4). Following a sensitivity analysis, it was found that by excluding arm 1 from the study of Celık et al. [33], WC exhibited a significant reduction following cornelian cherry supplementation (SMD = −0.53, CI: −1.05, −0.01, *p* = 0.05) (Figure S1A in Supplementary Materials). Nonetheless, the heterogeneity remained notably high (I^2^ = 76%, *p* = 0.002). Excluding arm 2 from the study conducted by Bayram et al. [29] led to a reduction in heterogeneity to below 50% (I^2^ = 30%, *p* = 0.22); however, the results were no more statistically significant (SMD = −0.19, CI: −0.48, 0.11, *p* = 0.22) (Figure S1B in Supplementary Materials).

#### 3.4.2. Effect of Cornelian Cherry Supplementation on TG, TC, LDL-C, and HDL-C Levels

**Figure 5:**
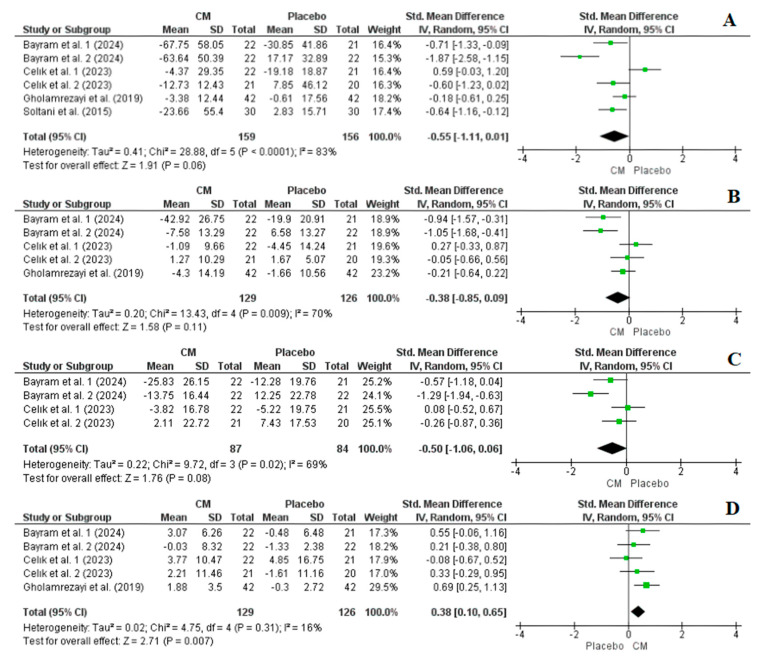
Forest plot representation of RCTs exploring the impact of cornelian cherry supplementation on blood lipid levels ((**A**): total triglycerides, (**B**): total cholesterol, (**C**): LDL-C, and (**D**): HDL-C) [28,29,30,33].

Four studies with a total of six arms, comprising 315 participants (159 in the intervention group and 156 in the placebo group), reported TG levels as an outcome measure. Overall, the results from the random-effects model indicated that cornelian cherry supplementation did not significantly reduce TG levels post-intervention (SMD = −0.55, CI: −1.11, 0.01, *p* = 0.06), with significant heterogeneity noted among the studies (I^2^ = 83%, *p* = 0.0001) (Figure 5). However, a sensitivity analysis revealed that excluding arm 1 from Celık et al. [33] led to a significant outcome favouring the cornelian cherry group over the control group (SMD = −0.76, CI: −1.26, −0.25, *p* = 0.003, I^2^ = 74%) (Figure S2A in Supplementary Materials).

Three studies with a total of five arms, comprising 255 participants (129 in the intervention group and 126 in the placebo group), reported TC and HDL-C levels as outcome measures. Overall, the results from the random-effects model indicated that cornelian cherry supplementation did not significantly reduce TC (SMD = −0.38, CI: −0.85, 0.09, *p* = 0.11, I^2^ = 70%), (Figure 5). However, HDL-C levels significantly increased post-intervention (SMD = 0.38, CI: 0.10, 0.65, *p* = 0.007, I^2^ = 16%) (Figure 5). Furthermore, a sensitivity analysis revealed that excluding arm 1 from Celık et al. [33] for the TC analysis led to a significant outcome favouring the cornelian cherry group over the control group (SMD = −0.54, CI: −1.02, −0.05, *p* = 0.03, I^2^ = 65%) (Figure S2B in Supplementary Materials).

Despite three studies with a total of five arms initially reporting LDL-C levels as an outcome measure, upon scrutinising the data, we opted to exclude those from Gholamrezayi et al. [30] due to apparent misreporting. Consequently, the final analysis included two studies with a total of four arms, comprising 171 participants (87 in the intervention group and 84 in the placebo group). The overall findings from the random-effects model indicated that cornelian cherry supplementation did not significantly reduce LDL-C levels post-intervention (SMD = −0.50, CI: −1.06, 0.06, *p* = 0.08, I^2^ = 69%) (Figure 5). A sensitivity analysis revealed that excluding arm 1 of the study conducted by Celık et al. [33] resulted in a significant outcome favouring the cornelian cherry group over the control group (SMD = −0.53, CI: −1.28, −0.11, *p* = 0.02, I^2^ = 62%) (Figure S2C in Supplementary Materials).

#### 3.4.3. Effect of Cornelian Cherry Supplementation on FBG, Insulin, HbA1c, and HOMA-IR

**Figure 6:**
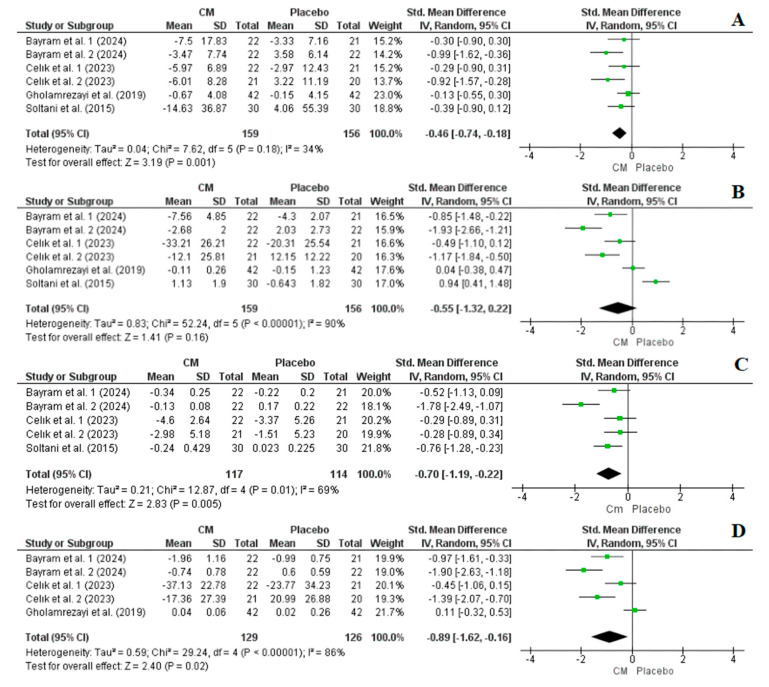
Forest plot representation of RCTs exploring the impact of cornelian cherry supplementation on glycaemic parameters ((**A**): fasting blood glucose, (**B**): insulin, (**C**): HbA1c, and (**D**): HOMA-IR) [28,29,30,33].

Four studies with a total of six arms, comprising 315 participants (159 in the intervention group and 156 in the placebo group), reported FBG and insulin levels as outcome measures. Overall, the results from the random-effects model indicated that cornelian cherry supplementation significantly reduced FBG (SMD = −0.46, CI: −0.74, −0.18, *p* = 0.001, I^2^ = 34%), while it did not significantly affect the insulin level (SMD = −0.55, CI: −1.32, 0.22, *p* = 0.16, I^2^ = 90%) (Figure 6). However, a sensitivity analysis revealed that excluding the study by Soltani et al. [28] resulted in a significant outcome favouring the cornelian cherry group over the control group regarding the insulin level (SMD = −0.85, CI: −1.52, −0.18, *p* = 0.01, I^2^ = 84%) (Figure S3A in Supplementary Materials).

Conversely, three studies with a total of five arms reported HbA1c and HOMA-IR as outcome measures, with a total of 231 participants (117 in the intervention group and 114 in the placebo group) and 255 participants (129 in the intervention group and 126 in the placebo group), respectively. Overall, the results from the random-effects model indicated that cornelian cherry supplementation significantly reduced HbA1c (SMD = −0.70, CI: −1.19, −0.22, *p* = 0.005, I^2^ = 69%) and HOMA-IR (SMD = −0.89, CI: −1.62, −0.16, *p* = 0.02, I^2^ = 86%) (Figure 6). Moreover, a sensitivity analysis revealed that excluding arm 2 from Bayram et al. [29] led to a decrease in heterogeneity, while preserving the observed effect for HbA1c (SMD = −0.48, CI: −0.78, −0.19, *p* = 0.001, I^2^ = 0%) (Figure S3B in Supplementary Materials). In the case of HOMA-IR, the sensitivity analysis did not result in a decrease in heterogeneity.

#### 3.4.4. Effect of Cornelian Cherry Supplementation on AST and ALT

**Figure 7:**
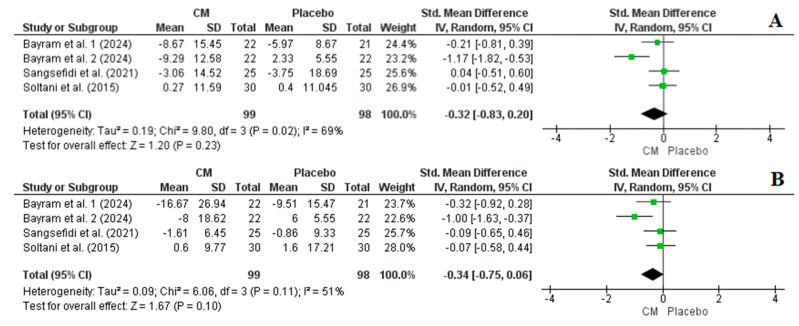
Forest plot representation of RCTs exploring the impact of cornelian cherry supplementation on liver parameters ((**A**): AST and (**B**): ALT) [28,29,31].

Three studies with a total of four arms, comprising 197 participants (99 in the intervention group and 98 in the placebo group), reported AST and ALT levels as outcome measures. Overall, the results from the random-effects model indicated that cornelian cherry supplementation did not significantly reduce AST (SMD = −0.34, CI: −0.75, 0.06, *p* = 0.10, I^2^ = 51%) and ALT (SMD = −0.32, CI: −0.83, 0.20, *p* = 0.23, I^2^ = 69%) levels (Figure 7). In both cases, the sensitivity analysis did not significantly affect the reported results.

#### 3.4.5. Effect of Cornelian Cherry Supplementation on SBP and DBP

Due to limited data availability, only one out of the six studies included in this meta-analysis provided information on systolic (SBP) and diastolic blood pressure (DBP) [32]. Consequently, this lack of data prevented us from conducting a meta-analysis on these outcomes. However, the findings from this study, which involved a 12-week administration of 20 mL/day of CM extract (equivalent to 2800 mg dried extract, containing 32 mg anthocyanins) among 50 patients with MAFLD, showed significant reductions in SBP and DBP. Specifically, there was a mean change in the intervention group of −8.63 ± 14.37 mmHg in SBP compared to 0.0 ± 12.67 mmHg in the placebo group (*p* = 0.02) and −8.62 ± 11.86 mmHg in DBP compared to 0.53 ± 8.53 mmHg (*p* = 0.003).

#### 3.4.6. Publication Bias

The evaluation of publication bias through a visual analysis of funnel plots alongside Egger’s test indicated the presence of publication bias in the meta-analysis of cornelian cherry supplementation for LDL-C (*p* = 0.003), FBG (*p* = 0.04), insulin (*p* = 0.01), HOMA-IR (*p* = 0.0001), AST (*p* = 0.03), and ALT (*p* = 0.03). However, the meta trim and fill analysis results did not reveal any publication bias except for FBG (*p* = 0.001). Egger’s linear regression test for other outcomes showed no presence of publication bias for TG (*p* = 0.26), TC (*p* = 0.53), HDL-C (*p* = 0.13), HbA1c (*p* = 0.19), BW (*p* = 0.39), BMI (*p* = 0.42), and WC (*p* = 0.42) (Table S1 in Supplementary Materials).

## 4. Discussion

Cardiometabolic diseases are the leading non-communicable diseases worldwide, representing the primary cause of global mortality and significantly affecting quality of life [35]. Obesity, dyslipidaemia, hypertension, insulin resistance, and high aminotransferase levels, among other factors, collectively contribute to an elevated risk of developing various cardiometabolic diseases like T2DM, cardiovascular disease, and metabolic-dysfunction-associated fatty liver disease [4,36]. Consequently, numerous dietary interventions have been developed to mitigate the modifiable risk factors associated with cardiometabolic diseases and enhance overall well-being [37,38,39]. Cornelian cherry has emerged as a promising dietary component with favourable health effects against cardiometabolic diseases, attracting significant attention within the scientific community. Within this framework, we conducted the first comprehensive review and meta-analysis of RCTs regarding its efficacy, evaluating the impact of supplementation with cornelian cherry on various cardiometabolic risk factors in adults, including anthropometric measurements, lipid profile, glycaemic parameters, blood pressure, and liver markers.

The results of the current meta-analysis suggested that supplementation with cornelian cherry may contribute to beneficial effects on certain risk factors linked to cardiometabolic diseases. Specifically, our findings supported a favourable impact of cornelian cherry supplementation on anthropometric measurements, lipid profile, and glycaemic parameters.

### 4.1. The Effect of Cornelian Cherry Supplementation on Anthropometric Measurements

Epidemiological data consistently link a high BW and BMI, respectively, with increased risks of medical complications and mortality. The distribution of body fat, particularly excess abdominal fat, plays a crucial role in predisposing individuals to obesity-related pathologies. To assess its level, WC is commonly employed as a surrogate marker due to its simplicity, correlation with abdominal fat mass (both subcutaneous and intra-abdominal), and association with the risk of cardiometabolic diseases [40]. According to our meta-analysis results, cornelian cherries may offer potentially beneficial effects on anthropometric parameters. Our findings, derived from a random-effects model, revealed that cornelian cherry supplementation significantly decreased BW and BMI, although it did not affect WC (Figure 4). However, subsequent sensitivity analysis revealed that excluding certain arms from individual studies led to variations in the results regarding WC. Notably, excluding one arm from a study resulted in a statistically significant reduction in WC with a high heterogeneity, while excluding another arm from a study reduced the heterogeneity to below 50%, although the results were no longer statistically significant.

The meta-analysis results align with other data from animal and human studies. For instance, a meta-analysis conducted by Park et al. [41] revealed a significant reduction in BMI following anthocyanin supplementation, while no significant changes in BW or WC were observed. Notably, anthocyanins decreased the BMI in non-obese individuals (BMI ≤ 25), but had no effect on obese individuals. Subgroup analyses indicated that doses below 300 mg/day of anthocyanins were more effective in reducing BMI, particularly over a four-week treatment period. Moreover, in a study examining the impact of anthocyanins from cornelian cherries on obesity in C57BL/6 mice fed with a high-fat diet, supplementation with anthocyanins (1 g/kg) for eight weeks led to a 24% decrease in weight gain and reduced lipid accumulation in the liver, as indicated by a significant decrease in liver triacylglycerol concentration [42]. Likewise, in a streptozotocin-induced diabetes mellitus model, administering 20 mg/kg of cornelian cherry extract resulted in reduced weight gain compared to the control group [43]. Similarly, in an animal model of obesity induced by ovariectomy, the oral administration of loganic acid, an iridoid from cornelian cherries, significantly curbed body weight gain, total fat accumulation, fatty hepatocyte deposition in the liver, and abdominal visceral fat tissue enlargement [44]. Treatment with the loganic acid of preadipocytes reduced the expression of key adipogenesis-related genes in a dose-dependent manner.

However, contrasting data exist in relation to our meta-analysis. The results of a separate meta-analysis indicated that anthocyanins did not significantly affect anthropometric and body composition parameters in humans [45]. No significant impacts on BMI and WC were observed in analyses of all subgroups following anthocyanins supplementation (regarding dosage, duration, design of the studies, etc). Moreover, Sangouni et al. [46] reported that, at the end of a 12-week intervention, there was no significant difference between the intervention (receiving 2800 mg/day of cornelian cherry fruit extract) and control groups regarding lipid accumulation product, an index calculated by WC and TG levels, which reflects lipid toxicity. Furthermore, in dyslipidaemic children and adolescents who received 50 g of cornelian cherry fruits twice a day for a period of six weeks, no significant difference in BMI was observed between the intervention and control groups at the end of the trial [47].

The reported contrasting results between different studies may be explained by various factors. Firstly, differences in study designs, such as the duration of the intervention, dosage of anthocyanins, and baseline anthocyanins’ intake of the studied population, could contribute to the contrarieties observed. For example, Daneshzad et al. showed that anthocyanin intake for more than 12 weeks of intervention was associated with an average of a 2.42 kg weight reduction and 0.75 kg/m^2^ decrease in BMI [48]. Secondly, differences in the formulations of cornelian cherry supplements used among the studies could impact the results. While certain studies reported the anthocyanin concentration of the supplemented extract, others only specified the quantity of the supplement, neglecting to mention the anthocyanin content. Additionally, the studies included various forms of cornelian cherry, such as liquid forms, extracts, and lyophilised powders. This diversity in the supplemented forms of cornelian cherry could have affected the availability of bioactive compounds, potentially influencing the outcomes [49].

### 4.2. The Effect of Cornelian Cherry Supplementation on Total Blood Lipid Levels

Dyslipidaemia can be characterised by elevated serum levels of TG, TC, and LDL-C or a reduced serum concentration of HDL-C. It is a well-established risk factor for coronary artery disease (CAD) [50]. The combined outcomes from the random-effects model revealed that, following cornelian cherry supplementation, there was a significant increase in HDL-C levels, although there was no significant decrease in post-intervention TG, TC, or LDL-C levels (Figure 5). However, a subsequent sensitivity analysis indicated that cornelian cherry supplementation might indeed lead to significant reductions in TG, LDL-C, and TC levels.

These findings align, in part, with those reported by Mohammadi et al. [18], who conducted a meta-analysis assessing the impact of cornelian cherry supplementation on the blood lipid profiles in animal rat models, concluding that cornelian cherry supplementation significantly lowered TG and TC levels, with a non-significant increase in HDL-C levels. Additionally, the daily ingestion of various amounts of cornelian cherry fruits (5, 10, and 15 g/d) over a period of 20 days in hamsters (*Mesocricetus auratus*) resulted in reductions in TC and LDL-C levels, while elevating HDL-C levels [16]. It was observed that the supplementation of 10 g/d cornelian cherry fruits demonstrated a more pronounced hypolipidemic effect compared to other doses (5 and 15 g/day).

Anthocyanins constitute the predominant compounds present in cornelian cherry. The predominant anthocyanins in cornelian cherry fruits include cyanidin-3-*O*-glucoside, cyanidin-3-*O*-robinobioside, and cyanidin-3-*O*-rutinoside (0.12–4.2 mg/g fw) [13]. In a meta-analysis conducted by Jang et al. [21], which included 41 studies, anthocyanin supplementation was found to significantly modulate various blood lipid markers, including reductions in TG levels and LDL-C levels, alongside an increase in HDL-C levels. Furthermore, in a subgroup analysis of the meta-analysis conducted by Daneshzad et al. [48], it was demonstrated that an anthocyanin supplementation dose of ≥300 mg/day decreased TC and LDL-C levels. In the studies included in our meta-analysis, however, the reported concentrations of anthocyanins ranged from 32 to 150 mg/day. This variability in dosage might account for the lack of significance in our results regarding TG, TC, and LDL-C levels before conducting the sensitivity analysis.

Several studies have explored the mechanisms underlying the positive impact of anthocyanins on blood lipid levels. In a double-blind, randomised, placebo-controlled trial involving 120 dyslipidaemic subjects aged 40–65 years, berry-derived anthocyanin supplements were administered twice daily for 12 weeks. It was shown that anthocyanin supplementation improved both LDL-C and HDL-C concentrations and enhanced cellular cholesterol efflux to serum, possibly through the inhibition of CETP [51]. Moreover, in an ApoE(−/−) mice model study, Wang et al. [52] showed that gut microbiota metabolised cyanidin-3-O-glucoside into protocatechuic acid (PCA), promoting cholesterol efflux from macrophages and enhancing the expression of ABCA1 and ABCG1 transporters. PCA also accelerated macrophage cholesterol efflux through the miRNA-10b-ABCA1/ABCG1 signalling cascade. Nevertheless, anthocyanins have the capacity to activate AMP-activated protein kinase, which, in turn, inhibits the synthesis of cholesterol and triglycerides by blocking HMG-CoA and acetyl-CoA carboxylase, respectively [48].

Conversely, cornelian cherry fruits are rich in iridoids, such as loganic acid, loganin, cornuside, sweroside, and catalposide. The total iridoid content of cornelian cherry fruits ranged between 0.87 and 4.94 mg/g fw [13]. The oral intake of cornelian cherry (100 mg/kg bodyweight) for 60 days in hypercholesterolemic rabbits led to a significant 44% decrease in serum triglyceride levels and prevented atheromatous changes in the thoracic aorta [53]. Additionally, cornelian cherry notably increased peroxisome proliferator-activated receptor-alpha (PPARα) protein expression in the liver, indicating enhanced fatty acid catabolism. The authors of the study suggested that iridoids were, in part, responsible for the observed effects [53]. In addition, these findings were corroborated in another study where the administration of loganic acid (20 mg/kg bodyweight) in hypercholesterolemic rabbits significantly decreased TG levels and increased HDL-C levels, also reducing the intima thickness and intima/media ratio in the thoracic aorta and decreasing plasma ox-LDL levels [54]. None of the studies included in our meta-analysis reported data regarding the iridoid content of cornelian cherry supplements administered to the study participants. Nevertheless, these findings collectively indicate that cornelian cherry fruits have the potential to impact blood lipid levels, although the supplementation form should be standardised to at least 300 mg of anthocyanins and/or 20 mg/kg bodyweight of iridoids (this statement should be experimentally evaluated).

### 4.3. The Effect of Cornelian Cherry Supplementation on Glycaemic Parameters

Elevated levels of glucose and insulin, both during fasting periods and after meals, as well as elevated HbA1c, are primary indicators of T2DM [55]. Among these risk factors, FBG demonstrated the strongest association with the onset of diabetes, followed by postprandial glucose (2hPG), HbA1c, HOMA-IR, and fasting insulin [56]. Our meta-analysis results demonstrated notable positive effects on several glycaemic indices, such as FBG, HbA1C, and HOMA-IR, following interventions with cornelian cherry fruits (Figure 6). Furthermore, a sensitivity analysis revealed a significant reduction in insulin levels after interventions with cornelian cherry fruits.

Several studies on animal models support our findings regarding the impact of cornelian cherry fruits and extracts on glycaemic parameters. For example, Jayaprakasam et al. [42] showed that supplementation with anthocyanins (1 g/kg) and ursolic acid (500 mg/kg), some of the most abundant bioactive compounds in cornelian cherries, in mice fed with a high-fat diet preserved islet architecture and insulin staining, suggesting anti-diabetic properties. Asgary et al. [57] found that administering 2 g/day of cornelian cherry fruits reduced FBG levels in alloxan-induced diabetic rats. Similarly, Capcarova et al. [58] observed decreased FBG levels in male Zucker diabetic fatty rats (fa/fa) consuming 1000 mg/kg of cornelian cherry fruits. Additionally, the administration of 20 mg/kg of cornelian cherry extract improved the glucose tolerance and lowered the blood glucose in rats with streptozotocin-induced diabetes mellitus [43]. Cornelian cherry has been shown to enhance the expression of insulin signalling genes in adipocytes, while increasing the expression of PPARγ, therefore alleviating insulin resistance and exerting a beneficial effect on cellular metabolism [59]. Furthermore, cornelian cherry fruits exhibited a potent inhibition of the exocrine enzymes involved in the breakdown of complex carbohydrates, specifically α-amylase and α-glucosidase, which catalyse the conversion of complex carbohydrates into readily digestible simple sugars [60].

In the meta-analysis conducted by Mao et al. [61], it was shown that anthocyanins at a median dose of 320 mg/day for a median intervention duration of eight weeks significantly reduced HbA1c and FBG, however, no statistically significant effects were observed on fasting insulin and HOMA-IR. Moreover, an umbrella review of systematic reviews and meta-analyses conducted by Sandoval-Ramírez et al. [62] showed that supplementation with 200–400 mg/day of anthocyanins significantly reduced FBG, HbA1c, and HOMA-IR in cardiometabolic participants. Mechanistically, anthocyanins act as α-glucosidase and α-amylase inhibitors, slowing carbohydrate digestion in the gut lumen [63]. Also, anthocyanins impact glucose absorption within the intestine, acting through active sodium-dependent and independent transport mechanisms mediated by sodium-glucose co-transporter 1 (SGLT1) and glucose transporter 2 (GLUT2) in both Caco-2 cells of the intestine and HepG2 cells [64]. Furthermore, anthocyanin consumption has been linked to enhanced glycogen synthesis and reduced gluconeogenesis in HepG2 cells and adipocytes [65]. These effects are attributed to the upregulation of PPARγ, a hormone involved in regulating adiponectin and the transcription of proteins crucial for the cellular uptake of glucose and fatty acids [66]. As a result, the upregulation of PPARγ leads to a decrease in fasting blood glucose levels.

As was mentioned above, ursolic acid (1.16 mg/g fw) and iridoids are some of the most abundant compounds found in cornelian cherry fruits [13]. For example, in a randomised, double-blind, placebo-controlled clinical trial involving 24 patients with untreated metabolic syndrome aged between 30 and 60 years, the effects of ursolic acid were evaluated by Ramírez-Rodríguez et al. [67]. The results showed, that after 12 weeks of ursolic acid intake (150 mg of ursolic acid/day), 50% of patients experienced a remission of metabolic syndrome, with significant improvements observed in FBG and insulin sensitivity. Moreover, the impact of treatment with ursolic acid on glycemia in hyperglycaemic rats and its underlying mechanisms in muscle tissue revealed that ursolic acid has a potent antihyperglycemic effect, increasing insulin vesicle translocation and secretion, as well as enhancing the glycogen content [68]. Ursolic acid stimulated glucose uptake in the muscle cells through classical insulin signalling pathways, involving the synthesis and translocation of GLUT4 to the plasma membrane. These effects were accompanied by an increased expression of GLUT4 mRNA, the activation of DNA transcription, and an enhanced presence of GLUT4 at the plasma membrane [68]. By contrast, a study which investigated the relationship between loganic acid content and the hypoglycaemic effects of cornelian cherry fruits showed that the tested extracts exhibited a strong inhibition of α-glucosidase activity [69], with the activity being correlated, in part, with the loganic acid present in the cornelian cherries. Further research showed that loganic acid administered orally to rats at 20 mg/kg for 14 days effectively restored the balance between antioxidant defence mechanisms and oxidative stress in leukocytes [70]. Although it did not significantly affect blood glucose levels, loganic acid notably improved the antioxidant status in leukocytes by increasing the levels of reduced glutathione and enhancing the activities of catalase, glutathione peroxidase, and glutathione reductase. Moreover, loganic acid demonstrated the ability to mitigate the formation and accumulation of glycation and oxidation protein products, as well as malondialdehyde derivatives in plasma [70]. The authors concluded that loganic acid holds promise as a potential therapeutic agent for alleviating the metabolic and functional disorders associated with diabetes, highlighting its potential for development as a component in new drug formulations.

### 4.4. The Effect of Cornelian Cherry Supplementation on Liver Parameters

ALT and AST are frequently evaluated markers of liver injury. Elevated levels of ALT and AST are significantly linked to an increased risk of developing cardiometabolic diseases [71,72]. Overall, the results of this meta-analysis indicated that cornelian cherry supplementation did not significantly reduce AST and ALT levels neither before nor after the sensitivity analysis (Figure 7).

Intriguingly, the scientific literature reported contrasting results. Although the animal studies reported indicated that anthocyanins may have beneficial effects in mitigating liver damage and improving liver function [73,74], human data did not support these results. A meta-analysis of 12 RCTs with a total number of 893 participants concluded that anthocyanins did not have any significant effects on ALT and AST [75]. Furthermore, another meta-analysis which included 15 RCTs and a total of 1028 participants showed that a significant reduction in AST was observed only in trials where individuals were supplemented with anthocyanin-rich products and in trials restricted to healthy subjects [76], indicating that anthocyanin supplementation may exert a more significant influence on decreasing AST levels in individuals without existing liver conditions.

### 4.5. The Effect of Cornelian Cherry Supplementation on Blood Pressure

Hypertension plays a central role in cardiometabolic diseases, showing the most compelling evidence for its causal link with cardiovascular disease [77]. However, only one out of six studies included in this meta-analysis evaluated the impact of cornelian cherry fruits on SBP and DBP, therefore, this scarcity of data prevented us from conducting a meta-analysis. Nevertheless, the findings from this study revealed that cornelian cherry supplementation resulted in significant reductions in both SBP and DBP in MAFLD subjects. The anthocyanin content of cornelian cherries may explain this effect, as dietary intake of anthocyanins has been linked to an 8% decrease in the risk of hypertension [78]. This effect may be due to anthocyanins’ ability to increase endothelial-derived nitric oxide levels by regulating endothelial NO synthase expression and activity [79]. Additionally, anthocyanins have demonstrated the ability to decrease the production of vasoconstricting molecules, including angiotensin II through ACE inhibition, endothelin 1, and thromboxanes via the inhibition of the cyclooxygenase pathway [80]. These actions promote blood vessel relaxation, ultimately resulting in decreased blood pressure [81].

### 4.6. Weight-Loss-Dependent and Independent Effects of Cornelian Cherry Supplementation

The question of how much the effects of cornelian cherry supplementation on lipid profiles, insulin sensitivity, blood pressure, and other cardiometabolic risk factors are mediated by weight loss versus being independent of body weight and adiposity is a critical one. Firstly, several studies have explored the relationship between weight loss and improvements in these risk factors, providing valuable context for interpreting the findings of the current meta-analysis. The meta-analysis conducted by Dattilo and Kris-Etherton showed that weight reduction is associated with significant improvements in lipid profiles, including decreases in TC, LDL-C, VLDL-C, and TG. Additionally, a modest increase in HDL-C was observed per kilogram of weight loss [82]. Further supporting this, Harder et al. demonstrated that a low-calorie diet resulting in significant weight loss led to marked improvements in the glycaemic control and lipid profiles in obese patients with T2DM [83]. This included reductions in FBG, fasting insulin, HbA1c, and LDL-C, alongside significant weight loss. Similarly, Zhou et al. found that weight loss in the first year following bariatric surgery was significantly correlated with long-term improvements in HbA1c and TG, highlighting the importance of weight loss for sustained glycaemic and metabolic control [84]. Lastly, Shinde et al. provided evidence from a large cohort study that sustained weight loss was associated with clinically meaningful improvements in glycaemic and metabolic parameters among individuals with T2DM. Greater weight loss percentages were linked to greater improvements in HbA1c and metabolic parameters, underscoring the role of weight management in managing T2DM and its complications [85].

Secondly, while these studies collectively highlight the significant role of weight loss in improving cardiometabolic risk factors in the context of a hypocaloric diet, it is also important to consider the potential direct effects of cornelian cherry supplementation on weight loss. In arm 2 of Bayram et al.’s and Gholamrezayi et al.’s studies, the only two studies where weight loss differences between the intervention and control groups were statistically significant, only arm 2 of Bayram et al.’s study showed improvements in the evaluated cardiometabolic parameters following further analysis. This potentially suggests a relationship between weight loss and improvements in cardiometabolic outcomes. However, the sensitivity analysis indicated a potential relationship only between arm 2 of Bayram et al.’s study and HOMA-IR, as excluding this arm from the meta-analysis nullified the significance of the effect. Apart from this, excluding arm 2 of Bayram et al.’s study did not alter any other reported results, suggesting that cornelian cherry supplementation may have a weight-loss-independent effect on cardiometabolic outcomes. Indeed, some of the bioactive compounds present in cornelian cherries, such as anthocyanins, loganic acid, and ursolic acid, have demonstrated beneficial effects on lipid metabolism, glucose regulation, and blood pressure independent of weight loss in preclinical studies [42,43,44]. However, distinguishing between weight-loss-dependent and -independent effects in the context of cornelian cherry supplementation still requires more rigorous studies.

### 4.7. Clinical Relevance of the Findings

The meta-analysis findings suggested that cornelian cherry supplementation has several beneficial effects on cardiometabolic risk factors, which are clinically relevant for managing and potentially reducing the risk of cardiovascular diseases and metabolic disorders. Incorporating cornelian cherry supplementation into weight management programs could enhance the effectiveness of these interventions, particularly for overweight or obese individuals. For individuals with T2DM or prediabetes, cornelian cherry supplementation could be an adjunct therapy for improving glycaemic control and insulin sensitivity, potentially reducing the need for medication or enhancing its effectiveness. The improvements in lipid profiles and potential reductions in blood pressure highlight the role of cornelian cherry supplementation in cardiovascular disease prevention and management. Therefore, this broad spectrum of beneficial effects on cardiometabolic risk factors suggests that cornelian cherry supplementation could contribute to overall metabolic health, reducing the risk of multiple chronic diseases. Moreover, dietary interventions, such as cornelian cherry supplementation, are often more accepted than pharmacological therapies due to their natural origin and the ability to incorporate them into a varied and balanced diet. This makes dietary strategies more sustainable and appealing to individuals compared to rigid medication regimens. However, the biomarkers investigated in this meta-analysis are considered to be soft endpoints. These markers, while valuable for understanding intermediate effects and physiological changes, do not always correlate with hard endpoints such as mortality or major cardiovascular events, thus, the evaluation of hard endpoints could provide a more comprehensive understanding of the long-term clinical relevance of cornelian cherry supplementation.

## 5. Limitations and Future Perspectives

To our knowledge, this is the first meta-analysis that evaluated the impact of cornelian cherry supplementation on different cardiometabolic risk factors in human RCTs, such as BW, BMI, and WC, TG, TC, LDL-C, HDL-C, SBP, DBP, FBG, insulin, HbA1c, HOMA-IR, AST, and ALT. Although the present meta-analysis offers valuable insights into the potential effects of cornelian cherry supplementation on various health parameters, some limitations should be considered.

### 5.1. Methodological Limitations

The primary limitation of the present meta-analysis lies in the applied methodology. The reliance on transforming data reported as medians and interquartile ranges (IQRs) into means and SDs assumes that the underlying distribution of the data is approximately normal. This transformation is a common approach in meta-analyses when individual study data are not reported in a format directly usable for pooled analyses. The formula used to estimate means and SDs from medians and IQRs, while widely cited in the literature, assumes normality and may not accurately capture the variability in the data, when the distribution is substantially non-normal. Furthermore, the transformation process itself introduces potential sources of error. Variability in the original data distribution, outliers, and the choice of transformation formula can all influence the accuracy of the estimated mean and SD. In addition, this transformation does not account for any asymmetry or kurtosis in the original data distribution, potentially leading to biased estimates of central tendency and dispersion. Therefore, this limitation has implications for the robustness and reliability of the meta-analysis results. Biased estimates of means and SDs derived from transformed data can affect the precision and accuracy of effect size estimates and may impact the overall conclusions drawn from meta-analyses. Moreover, the assumption of normality underlying the transformation may not be justified in all cases, particularly in studies with small sample sizes (as was the case with two of included studies) or non-standard populations.

### 5.2. Study-Related Limitations

The total sample size across the included studies was relatively small (415 participants, predominantly females (~70%)), which may impact the generalisability of the findings and statistical power of the analyses. Also, there were considerable variations in the dosages and durations of cornelian cherry supplementation across the included studies. Diverse dosages and durations could lead to inconsistent outcomes and dilute the ability to draw definitive conclusions about the optimal intervention. Moreover, the studies included participants with different health conditions, such as MAFLD, T2DM, insulin resistance, and postmenopausal status. Variability in participant characteristics could introduce confounding variables that affect the outcomes. Also, in some studies, the observed improvements in cardiometabolic risk factors might be partially attributed to weight loss, which is inherently correlated with improvements in lipid levels and glycaemic parameters. This suggests that the beneficial effects seen with cornelian cherry supplementation could, in part, be due to its influence on body weight, rather than direct effects on these risk factors. Distinguishing between the weight-loss-dependent and -independent effects of cornelian cherry supplementation remains a challenge and highlights the need for more controlled studies in this area. Lastly, the risk of bias assessment using the RoB 2 tool revealed concerns and a high risk of bias across several domains in some studies. This could potentially affect the reliability and validity of the reported results.

### 5.3. Geographic and Supplement Quality Limitations

Most included studies were conducted in specific geographic regions (Iran and Turkey), which may limit the generalisability of the findings to other populations with different dietary habits, lifestyles, and genetic backgrounds. Additionally, dietary supplements often lack the same reproducible quality and dose consistency as pharmaceuticals. This variability in supplement composition can impact the consistency of the observed effects across different studies.

### 5.4. Future Research Directions

Addressing the presented limitations in future research could enhance the robustness and validity of the findings regarding the potential benefits of cornelian cherry supplementation. Future studies should focus on improving of the generalisability of the findings by including participants with various demographic backgrounds, including different ethnicities, geographical regions, socioeconomic statuses, and background pathologies, therefore providing a more comprehensive understanding of how cornelian cherry supplementation affects different population groups, regardless of their health status. Moreover, utilising a standardised cornelian cherry supplement with a consistent dosage of at least 300 mg of anthocyanins would allow for more accurate comparisons across studies and facilitate meta-analyses, thus ensuring consistency in the composition and potency of the supplement, minimising variability in the observed effects. In addition to anthocyanins, future research should evaluate the presence and concentration of other bioactive compounds in the final supplement, such as loganic and ursolic acids, which showed beneficial results in preclinical and some clinical studies. This comprehensive approach would provide insights into the potential synergistic effects of these compounds on health outcomes. Furthermore, studies with a longer duration (≥12 weeks) would allow researchers to assess the long-term effects of cornelian cherry supplementation on various health parameters. Lastly, future studies should prioritise rigorous study design and implementation to minimise the risk of bias and enhance internal validity. This approach includes employing robust randomisation procedures, blinding participants and researchers, addressing missing data comprehensively, and transparently reporting study protocols and outcomes. Utilising validated outcome measures and standardised data collection methods would further enhance the reliability and reproducibility of the findings. By addressing these key areas in future research, researchers can advance our understanding of the potential health benefits of cornelian cherry supplementation and provide evidence-based recommendations for its use in clinical practice.

## 6. Conclusions

This meta-analysis included six RCTs with a total number of 415 participants, suggesting that cornelian cherry supplementation might have beneficial effects on anthropometrical parameters (BW and BMI), lipid profiles (TG, TC, LDL-C, and HDL-C), and glycaemic parameters (FBG, insulin, HbA1c, and HOMA-IR). However, the inherent limitation of the meta-analysis method, coupled with the constrains of the included studies, such as the small sample size, heterogeneity in dosages and durations, variability in participant characteristics, risk of bias, and limited generalisability to diverse populations and health conditions, underscore the need for further investigation. Future research should aim to address these limitations by including larger and more diverse study populations, utilising standardised supplement dosages, evaluating additional bioactive compounds, extending supplementation periods, and enhancing the study design for internal validity. By doing so, the reliability and applicability of findings related to cornelian cherry supplementation can be improved, while providing more robust evidence for its potential health benefits.

## Figures and Tables

**Table 1:** The PICOS framework summarising the rationale behind the study, outlining the criteria for selecting studies.

Criteria	Description
Population	Adult participants (aged ≥ 18 years), excluding pregnant individuals, whether healthy or otherwise
Intervention	Supplementation with cornelian cherry fruits/powder/extract
Comparison	Placebo
Study	Evaluation of the effects of cornelian cherry on cardiometabolic risk factors, including BW, BMI, WC, TG, TC, LDL-C, HDL-C, SBP, DBP, FBG, insulin, HbA1c, HOMA-IR, AST, and ALT
Outcome	Randomised controlled trials with either a crossover or parallel trial design, lasting at least ≥2 weeks

Abbreviations: Body weight (BW), body mass index (BMI), waist circumference (WC), triglycerides (TG), total cholesterol (TC), low-density lipoprotein cholesterol (LDL-C), high-density lipoprotein cholesterol (HDL-C), systolic blood pressure (SBP), diastolic blood pressure (DBP), fasting blood glucose (FBG), glycated haemoglobin (HbA1c), homeostatic model assessment of insulin resistance (HOMA-IR), aspartate aminotransferase (AST), and alanine aminotransferase (ALT).

**Table 2:** Characteristics of included RCTs evaluating supplementation with cornelian cherry fruit/powder/extract on selected cardiometabolic outcomes.

Study (Year), Country	Study Design	Participants	Intervention/d	Control	Type of Intervention	Duration	Total Sample (Intervention/Placebo) Sex Distribution (M/F)	Measured Outcomes
Soltani et al. (2015), Iran [28]	Parallel, double-blinded, randomised clinical trial	T2DM	500 mg CM extract (150 mg anthocyanins)	Placebo	Extract	6 wk	60 (30/30) (39/21)	↔ BMI, ↔ FBG, ↓ Insulin, ↓ HbA1c, ↓ TG, ↔ ALT, ↔ AST
Gholamrezayi et al. (2019), Iran [30]	Parallel, double-blinded, randomised clinical trial	Postmenopausal women	900 mg CM extract	Placebo	Extract	8 wk	84 (42/42) (0/84)	↓ BW, ↓ BMI, ↓ WC, ↔ FBG, ↔ Insulin, ↔ HOMA-IR, ↔ TG, ↔ LDL-C, ↑ HDL-C, ↔ TC
Sangsefidi et al. (2021), Iran [31]	Parallel, double-blinded, randomised clinical trial	MAFLD	20 mL CM extract (2800 mg dried extract, 32 mg anthocyanins)	Placebo	Extract	12 wk	50 (25/25) (23/27)	↔ ALT, ↔ AST
Celık et al. (2023), Turkey [33]	Parallel, randomised controlled trial	Insulin resistance	20 g lyophilised dried CM powder	Placebo	Powder	12 wk	84 (43/41) (0/84)	↔ BW, ↔ BMI, ↔ WC, ↓ FBG, ↓ HbA1c, ↓ Insulin, ↓ HOMA-IR, ↓ TC, ↔ LDL-C, ↔ HDL-C, ↓ TG
Yarhosseini et al. (2023), Iran [32]	Parallel, double-blinded, randomised clinical trial	MAFLD	20 mL CM extract (2800 mg dried extract, 32 mg anthocyanins)	Placebo	Extract	12 wk	50 (25/25) (23/27)	↔ BW, ↔ WC, ↓ SBP, ↓ DBP
Bayram et al. (2024), Turkey [29]	Parallel, single-blinded, randomised controlled trial	MAFLD	30 g lyophilised dried CM powder	Placebo	Powder	8 wk	87 (44/43) (40/47)	↓ BW, ↓ BMI, ↓ WC, ↓ FBG, ↓ Insulin, ↓ HOMA-IR, ↓ HbA1c, ↓ AST, ↓ ALT, ↓ TG, HDL-C, ↓ LDL-C, ↓ TC

Abbreviations: *Cornus mas* (CM), metabolic-dysfunction-associated fatty liver disease (MAFLD), type 2 diabetes mellitus (T2DM), body weight (BW), body mass index (BMI), waist circumference (WC), triglycerides (TG), total cholesterol (TC), low-density lipoprotein cholesterol (LDL-C), high-density lipoprotein cholesterol (HDL-C), systolic blood pressure (SBP), diastolic blood pressure (DBP), fasting blood glucose (FBG), glycated haemoglobin (HbA1c), homeostatic model assessment of insulin resistance (HOMA-IR), aspartate aminotransferase (AST), and alanine aminotransferase (ALT). ↓, ↑ presence of an effect (*p* < 0.05), ↔ no effect.

## Data Availability

The original contributions presented in the study are included in the article/supplementary material, further inquiries can be directed to the corresponding author/s.

## Supplementary Materials

The following supporting information can be downloaded at: https://www.mdpi.com/article/10.3390/nu16132173/s1, Figure S1: Forest plot representation of sensitivity analysis exploring the impact of cornelian cherry supplementation on waist circumference; Figure S2: Forest plot representation of sensitivity analysis exploring the impact of cornelian cherry supplementation on blood lipid levels; Figure S3: Forest plot representation of sensitivity analysis exploring the impact of cornelian cherry supplementation on glycaemic parameters; Table S1: The *p*-values from Egger’s linear regression test for each outcome indicate the significance of the funnel plot asymmetry for each respective outcome.
