# Supplementary material for: The Impact of Cornelian Cherry (Cornus mas L.) on Cardiometabolic Risk Factors: A Meta-Analysis of Randomised Controlled Trials
Source: Nutrients. 2024 Jul 8;16(13):2173. doi: 10.3390/nu16132173 (PMC11243109; doi:10.3390/nu16132173)
Supplement: Supplementary file 1 [file nutrients-16-02173-s001.zip › nutrients-3065466-supplementary.pdf]

A

Study or Subgroup	CM Mean	CM SD	CM Total	Placebo Mean	Placebo SD	Placebo Total	Weight	Std. Mean Difference IV, Random, 95% CI
Bayram et al. 1 (2024)	-5.42	3.37	22	-4	3.18	21	19.6%	-0.43 [-1.03, 0.18]
Bayram et al. 2 (2024)	-1.25	0.99	22	0.33	0.79	22	18.0%	-1.73 [-2.43, -1.03]
Celık et al. 1 (2023)	-6.17	2.41	22	-6.91	2.94	21	0.0%	0.27 [-0.33, 0.87]
Celık et al. 2 (2023)	-1.69	3.25	21	-1.66	2.95	20	19.5%	-0.01 [-0.62, 0.60]
Gholamrezayi et al. (2019)	-2.21	2.46	42	-0.8	2.79	42	22.5%	-0.53 [-0.97, -0.10]
Yarhosseini et al. (2023)	0.8	3.3	25	1	2.7	25	20.5%	-0.07 [-0.62, 0.49]
**Total (95% CI)**			**132**			**130**	**100.0%**	**-0.53 [-1.05, -0.01]**

Heterogeneity: Tau² = 0.27; Chi² = 16.72, df = 4 (P = 0.002); I² = 76%
Test for overall effect: Z = 1.98 (P = 0.05)

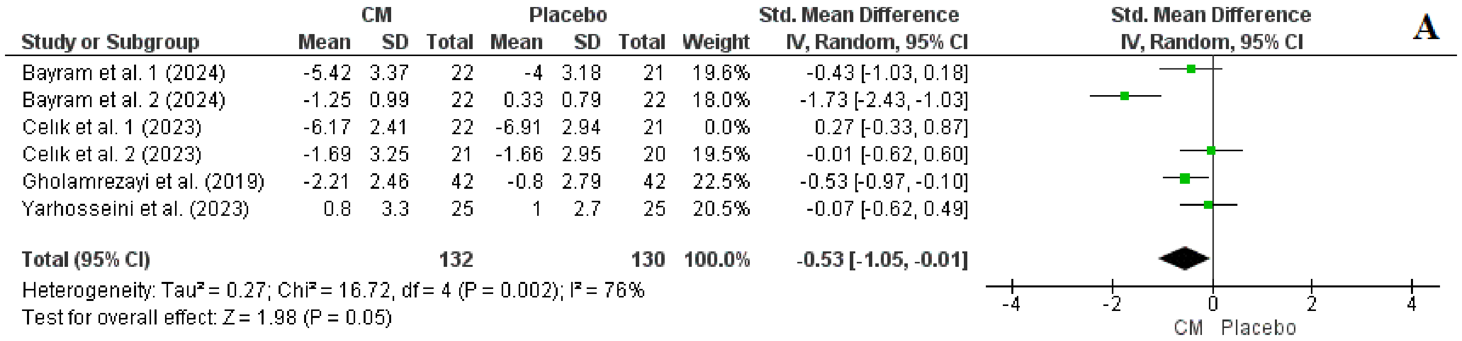

B

Study or Subgroup	CM Mean	CM SD	CM Total	Placebo Mean	Placebo SD	Placebo Total	Weight	Std. Mean Difference IV, Random, 95% CI
Bayram et al. 1 (2024)	-5.42	3.37	22	-4	3.18	21	17.6%	-0.43 [-1.03, 0.18]
Bayram et al. 2 (2024)	-1.25	0.99	22	0.33	0.79	22	0.0%	-1.73 [-2.43, -1.03]
Celık et al. 1 (2023)	-6.17	2.41	22	-6.91	2.94	21	17.8%	0.27 [-0.33, 0.87]
Celık et al. 2 (2023)	-1.69	3.25	21	-1.66	2.95	20	17.3%	-0.01 [-0.62, 0.60]
Gholamrezayi et al. (2019)	-2.21	2.46	42	-0.8	2.79	42	27.3%	-0.53 [-0.97, -0.10]
Yarhosseini et al. (2023)	0.8	3.3	25	1	2.7	25	20.0%	-0.07 [-0.62, 0.49]
**Total (95% CI)**			**132**			**129**	**100.0%**	**-0.19 [-0.48, 0.11]**

Heterogeneity: Tau² = 0.03; Chi² = 5.71, df = 4 (P = 0.22); I² = 30%
Test for overall effect: Z = 1.24 (P = 0.22)

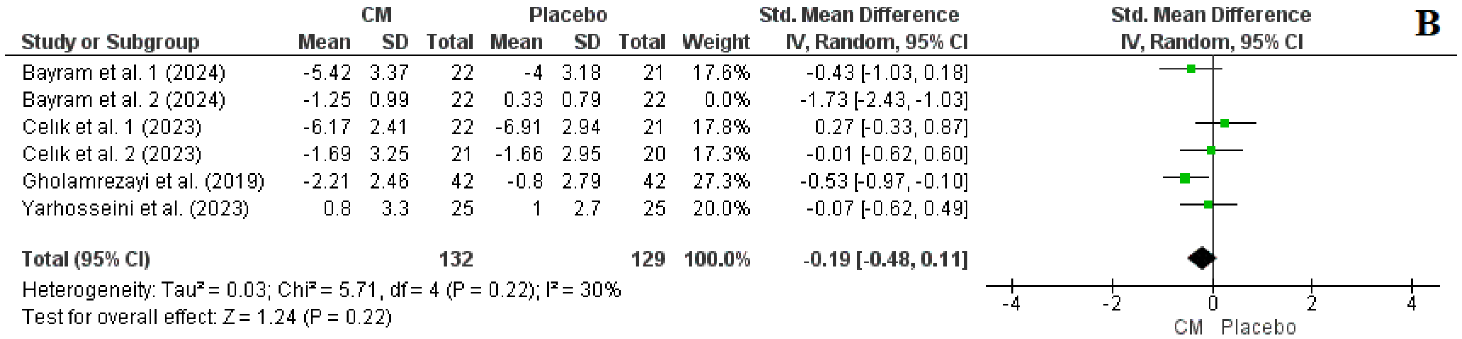

Figure S1. Forest plot representation of sensitivity analysis exploring the impact of cornelian cherry supplementation on waist circumference (**A**: exclusion of arm 1 from Celık et al. study (1), **B**: exclusion of arm 2 from Bayram et al. study (2)).

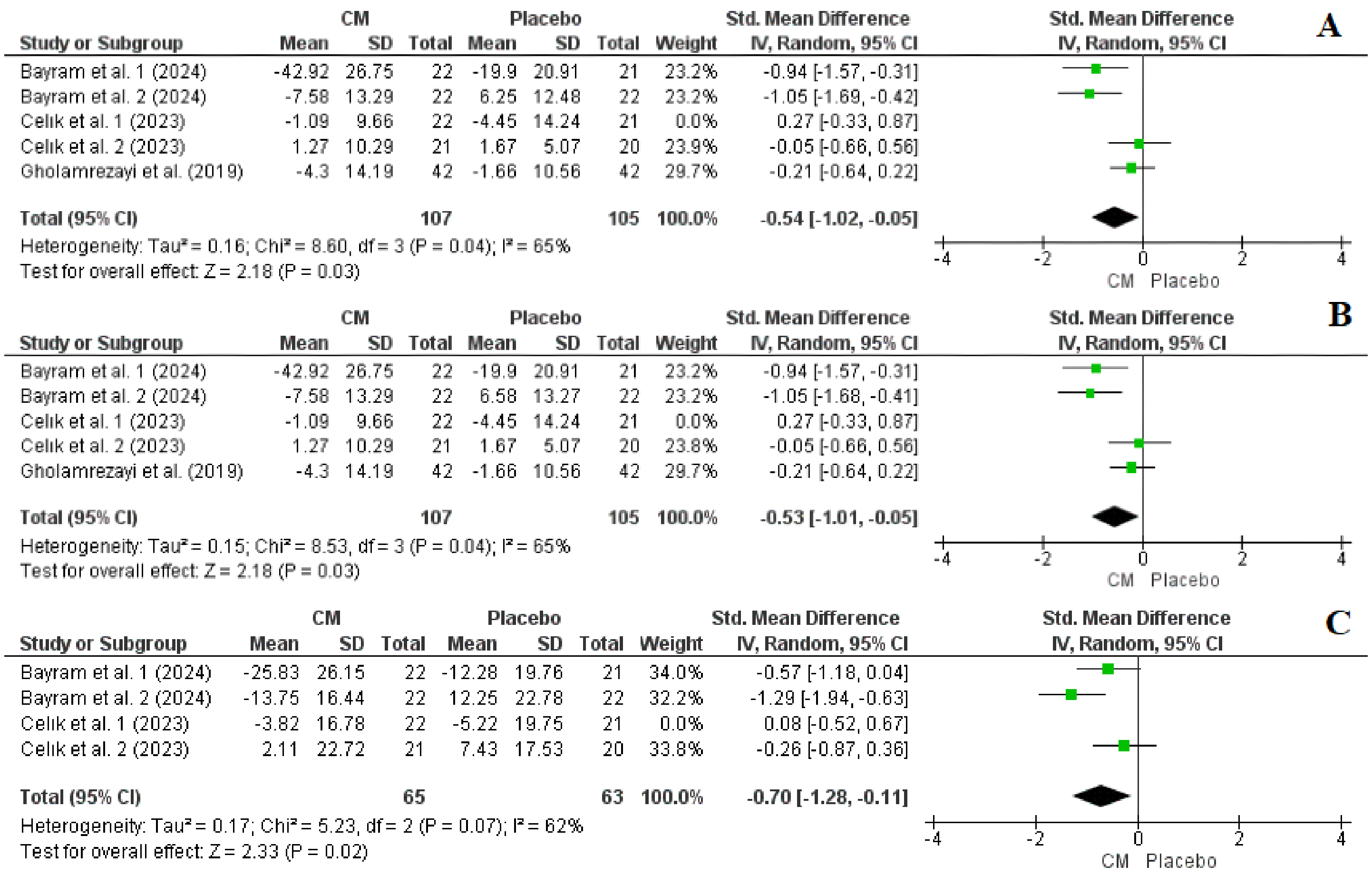

A

Study or Subgroup	CM Mean	CM SD	CM Total	Placebo Mean	Placebo SD	Placebo Total	Weight	Std. Mean Difference IV, Random, 95% CI
Bayram et al. 1 (2024)	-42.92	26.75	22	-19.9	20.91	21	23.2%	-0.94 [-1.57, -0.31]
Bayram et al. 2 (2024)	-7.58	13.29	22	6.25	12.48	22	23.2%	-1.05 [-1.69, -0.42]
Celık et al. 1 (2023)	-1.09	9.66	22	-4.45	14.24	21	0.0%	0.27 [-0.33, 0.87]
Celık et al. 2 (2023)	1.27	10.29	21	1.67	5.07	20	23.9%	-0.05 [-0.66, 0.56]
Gholamrezayi et al. (2019)	-4.3	14.19	42	-1.66	10.56	42	29.7%	-0.21 [-0.64, 0.22]
**Total (95% CI)**			**107**			**105**	**100.0%**	**-0.54 [-1.02, -0.05]**

Heterogeneity: Tau² = 0.16; Chi² = 8.60, df = 3 (P = 0.04); I² = 65%
Test for overall effect: Z = 2.18 (P = 0.03)

B

Study or Subgroup	CM Mean	CM SD	CM Total	Placebo Mean	Placebo SD	Placebo Total	Weight	Std. Mean Difference IV, Random, 95% CI
Bayram et al. 1 (2024)	-42.92	26.75	22	-19.9	20.91	21	23.2%	-0.94 [-1.57, -0.31]
Bayram et al. 2 (2024)	-7.58	13.29	22	6.58	13.27	22	23.2%	-1.05 [-1.68, -0.41]
Celık et al. 1 (2023)	-1.09	9.66	22	-4.45	14.24	21	0.0%	0.27 [-0.33, 0.87]
Celık et al. 2 (2023)	1.27	10.29	21	1.67	5.07	20	23.8%	-0.05 [-0.66, 0.56]
Gholamrezayi et al. (2019)	-4.3	14.19	42	-1.66	10.56	42	29.7%	-0.21 [-0.64, 0.22]
**Total (95% CI)**			**107**			**105**	**100.0%**	**-0.53 [-1.01, -0.05]**

Heterogeneity: Tau² = 0.15; Chi² = 8.53, df = 3 (P = 0.04); I² = 65%
Test for overall effect: Z = 2.18 (P = 0.03)

C

Study or Subgroup	CM Mean	CM SD	CM Total	Placebo Mean	Placebo SD	Placebo Total	Weight	Std. Mean Difference IV, Random, 95% CI
Bayram et al. 1 (2024)	-25.83	26.15	22	-12.28	19.76	21	34.0%	-0.57 [-1.18, 0.04]
Bayram et al. 2 (2024)	-13.75	16.44	22	12.25	22.78	22	32.2%	-1.29 [-1.94, -0.63]
Celık et al. 1 (2023)	-3.82	16.78	22	-5.22	19.75	21	0.0%	0.08 [-0.52, 0.67]
Celık et al. 2 (2023)	2.11	22.72	21	7.43	17.53	20	33.8%	-0.26 [-0.87, 0.36]
**Total (95% CI)**			**65**			**63**	**100.0%**	**-0.70 [-1.28, -0.11]**

Heterogeneity: Tau² = 0.17; Chi² = 5.23, df = 2 (P = 0.07); I² = 62%
Test for overall effect: Z = 2.33 (P = 0.02)

Figure S2. Forest plot representation of sensitivity analysis exploring the impact of cornelian cherry supplementation on blood lipid levels (A: Total triglycerides, B: Total cholesterol, C: LDL-C).

A

Study or Subgroup	CM Mean	CM SD	CM Total	Placebo Mean	Placebo SD	Placebo Total	Weight	Std. Mean Difference IV, Random, 95% CI
Bayram et al. 1 (2024)	-7.56	4.85	22	-4.3	2.07	21	19.9%	-0.85 [-1.48, -0.22]
Bayram et al. 2 (2024)	-2.68	2	22	2.03	2.73	22	18.7%	-1.93 [-2.66, -1.21]
Celık et al. 1 (2023)	-33.21	26.21	22	-20.31	25.54	21	20.1%	-0.49 [-1.10, 0.12]
Celık et al. 2 (2023)	-12.1	25.81	21	12.15	12.22	20	19.4%	-1.17 [-1.84, -0.50]
Gholamrezayi et al. (2019)	-0.11	0.26	42	-0.15	1.23	42	21.9%	0.04 [-0.38, 0.47]
Soltani et al. (2015)	1.13	1.9	30	-0.643	1.82	30	0.0%	0.94 [0.41, 1.48]
**Total (95% CI)**			**129**			**126**	**100.0%**	**-0.85 [-1.52, -0.18]**

Heterogeneity: $Tau^2 = 0.48$; $Chi^2 = 25.07$, df = 4 (P < 0.0001); $I^2 = 84\%$
Test for overall effect: Z = 2.48 (P = 0.01)

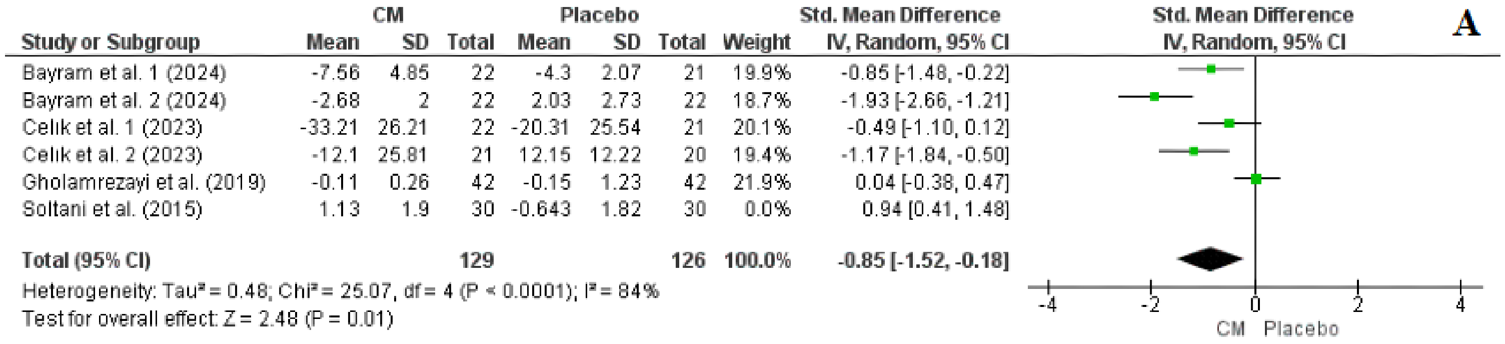

B

Study or Subgroup	CM Mean	CM SD	CM Total	Placebo Mean	Placebo SD	Placebo Total	Weight	Std. Mean Difference IV, Random, 95% CI
Bayram et al. 1 (2024)	-0.34	0.25	22	-0.22	0.2	21	23.0%	-0.52 [-1.13, 0.09]
Bayram et al. 2 (2024)	-0.13	0.08	22	0.17	0.22	22	0.0%	-1.78 [-2.49, -1.07]
Celık et al. 1 (2023)	-4.6	2.64	22	-3.37	5.26	21	23.6%	-0.29 [-0.89, 0.31]
Celık et al. 2 (2023)	-2.98	5.18	21	-1.51	5.23	20	22.5%	-0.28 [-0.89, 0.34]
Soltani et al. (2015)	-0.24	0.429	30	0.023	0.225	30	30.9%	-0.76 [-1.28, -0.23]
**Total (95% CI)**			**95**			**92**	**100.0%**	**-0.48 [-0.78, -0.19]**

Heterogeneity: $Tau^2 = 0.00$; $Chi^2 = 1.88$, df = 3 (P = 0.60); $I^2 = 0\%$
Test for overall effect: Z = 3.25 (P = 0.001)

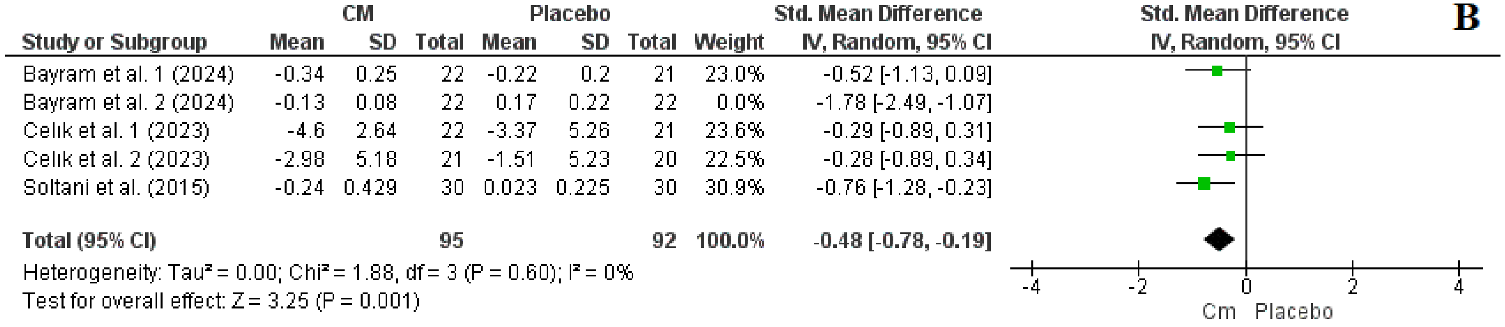

Figure S3. Forest plot representation of sensitivity analysis exploring the impact of cornelian cherry supplementation on glycaemic parameters (A: Insulin levels, B: HbA1c).

Table S1. The p-values from Egger's linear regression test for each outcome indicate the significance of the funnel plot asymmetry for each respective outcome.

**Outcome**	**p-value**	**Meta trim and fill analysis p-value**
TG	0.26	-
TC	0.53	-
LDL	0.003	0.08
HDL	0.13	-
FBG	0.04	0.001
Insulin	0.01	0.17
HbA1c	0.19	-
HOMA-IR	0.0001	0.30
AST	0.03	0.09
ALT	0.03	0.24
BW	0.39	-
BMI	0.42	-
WC	0.42	-
